# Transcriptional modulation of squalene synthase genes in barley treated with PGPR

**DOI:** 10.3389/fpls.2015.00672

**Published:** 2015-09-01

**Authors:** Anam Yousaf, Abdul Qadir, Tehmina Anjum, Aqeel Ahmad

**Affiliations:** ^1^College of Earth and Environmental Sciences, University of the Punjab, LahorePakistan; ^2^Institute of Agricultural Sciences, University of the Punjab, LahorePakistan

**Keywords:** squalene synthase, RT-PCR, phytosterol, transcription, genes, modulation, rhizospheric

## Abstract

Phytosterol contents and food quality of plant produce is directly associated with transcription of gene squalene synthase (SS). In current study, barley plants were treated with different rhizobacterial strains under semi controlled (27 ± 3°C) greenhouse conditions in order to modulate expression of SS gene. Plant samples were analyzed through semi-quantitative PCR to evaluate effect of rhizobacterial application on transcriptional status of SS. Results revealed that among four SS genes (i.e., SSA, SS1, SS2, and SS3), the most expressive gene was SSA; while, SS2 was screened out as the second best induced gene due to *Acetobacter aceti*. The most efficient bacterial strain which recorded maximum gene expression was *A. aceti* AC8. Moreover, AC7 was reported as the least efficient bacterial species for inducing SS gene expression. AC8 enhanced the share of SSA and SS2 up to 43 and 31%, respectively. The study also described ribosomal sequence of the most efficient bacterial strain AC8, which was used to determine its phylogenetic relationships with other microbial strains. The study would be helpful to improve quality of plant produce by modulating transcription of SS genes.

## Introduction

Phytosterols are an important content of human food, which control lipid and cholesterol absorption and prevent heart diseases. Consumption of phytosterol rich food can reduce heart and circulatory system disorders significantly. Previous studies that carried out to evaluate food commodities for their phytosterol and other dietary contents ranked barley among the first crops in that index (Carr et al., 2010). It was recommended that barley should be consumed as staple food item to control obesity and hyperlipidemia. This recommendation was made on the basis of more phytosterols in barley than other staple food crops. Now, it is important to enhance constitutive phytosterols of barley using some environmentally safe measures. This goal can be achieved through a number of techniques including genetic engineering, plant breeding and modulation of transcriptional profile through biological agents. Genetically engineered plants and breeding programs have not been the point of interest for many experts due to their environmental hazards and time consuming operations. Using biological entity to modulate transcriptional profile has always been an encouraged technique due to its low cost, easy application, high efficiency, and environmentally safe nature. Therefore, application of non-pathogenic microbes has been selected to enhance phytosterol contents in barley seeds.

Squalene is a key precursor of phytosterol synthesis in plant cells, which play an important role in production of phytosterols. Quantities of squalene and phytosterols have been found directly associated with transcription of squalene synthase (SS) gene (Lee et al., 2004). Thus, SS is a key factor in production of phytosterol and its transcription is directly related to the production of phytosterols by plants. Transcriptional rates of SS indicate the quantity of phytosterol at any developmental stage of plant. Rate of transcription of SS has been used frequently to determine quantity of phytosterol in plants (Nguyen et al., 2013). Moreover, studies based upon transcriptional rates of genes are considered more reliable than studies based on other techniques. Current investigation determines the induction of phytosterol contents in barley seeds under the activity of non-pathogenic bacterial strains. For this purpose, transcriptional rate of SS has been used as a key parameter to evaluate this induction. The current study will be a remarkable contribution in improving human food.

## Materials and Methods

Selected staple food crop ‘barley’ was grown in greenhouse of Institute of Agricultural Sciences, University of the Punjab Lahore, Pakistan under semi controlled conditions (27 ± 3°C). Barley was treated with different strains of bacteria *Acetobacter aceti* (i.e., AC1, AC2, AC3…. AC8). Therefore, inoculum of each bacterial strain was separately prepared from 16 h old bacterial cultures. Bacterial cells were collected through centrifugation at 5000 rpm and then its inoculum was prepared in distilled sterilized water. Inoculum concentration of each bacterial strain was adjusted to 1 × 10^6^ bacterial cells/mL. Moreover, 100 mL of bacterial inoculum was added into soil of respective pots (of 12 inches diameter), containing three barley plants in each pot. Each treatment was replicated thrice, while each replicate was consisted upon 50 pots. Plants were watered when needed and seeds of mature plants were randomly harvested prior to their processing for gene expressions analysis.

### Reverse Transcriptase (PCR)

Barley seeds were collected and immediately preserved in liquid nitrogen in order to minimize the molecular reactions. Then, 0.5 g of material was crushed in liquid nitrogen to extract RNA. RiboEx (TM) of “biomol” was used to isolate RNA and M-MLV kit of “Enzynomics” was further used to synthesize cDNA. DNAse I (Roche Diagnostics Mannheim, Germany) was added into RNA samples at 37°C for 30 min and then isolated RNA was checked spectrophotometrically for its absorbance at 260 nm. Specific gene primers were designed and used against PCR amplifications as mentioned in (**Table 1**). Specific designed primer set was used to target Gene α-Tubulin as internal control (housekeeping) gene [F]: TGAACAACTCATAAGTGGCAAAG; and [R]: TCCAGCAGAAGTGACCCAAGAC (Xu and Shi, 2006). All the selected genes were amplified separately at their optimum thermal cyclic conditions and then 5 μL of amplicon was loaded in a similar well on 1% agarose gel. Agarose gel was further analyzed using GELANALYZER (Lazar, Hungary) and statistical analysis was also carried out through DSAASTAT (Onofri, Italy).

**Table 1:** Sequence of primer sets used to target specific Squalene Synthase (SS) genes in barley.

Gene	Abbreviation	Primer details (5′……3′)
Squalene Synthase A	SSA	AAG ATT TCT ATC CGT TGT TGA AGC AAA CTA GGA ATT TGC TTG TGC ATC
Squalene Synthase 1	SS1	ACA AAA GTA CCG AGG GTT GAT GA TAT ATA TCT GTT TCG CCA AGC ACA A
Squalene Synthase 2	SS2	TGCAAATCAAGCAATCTTATCTAAGA TTT GCA AAA CCC AAT CAC AGG CT
Squalene Synthase 3	SS3	TAC CAA TAG TGA TGG CTT TTC ATT TTT GCA AAA CCC AAT CAC AGG CT

#### Identification of the best bacterial strain

Initially, identification of the best performing bacterial strain was carried out through biochemical means and bacterial strain was identified as *A.aceti*. Then ribotyping of *A. aceti* was performed to confirm the identification of bacterial species on molecular basis. Thus, 16S sequence of the bacteria was amplified in a gene specific PCR reaction with 0.5 mM of each primers F: TTT TCG GAT TGT AAA GCA CTT TC and R: TTC TCA CGA CAC GCG CTT. Moreover, PCR mixture consisted upon MgCl_2_ (1.5 mM), 0.8 mM deoxynucleoside triphosphate (dNTP) mixture, Taq DNA polymerase (0.6 U) and genomic DNA of the bacterial strain (20 ng) in a total volume of 25 mL. Initial denaturation was carried out at 94 °C for 5 min, and final extension at 72°C for 10 min. Thermocyclic conditions provided to PCR reaction were, denaturation at 94°C for 1 min, annealing at 58°C for 30 s and extension at 72°C for 1 min.

#### Verification of Elevated Phytosterol Contents

Induction of phytosterol contents was also confirmed using biochemical analysis of barley seeds. For this purpose, method of Mo et al. (2013) was followed. Briefly, seeds of treated as well as control plants were harvested and their endosperms were crushed separately. Preweighed (1 g) of crushed seed material was extracted with 10 mL of 80% ethanol. The extracts were dried up to 0.2 mL volume and added up with 10 μL of internal standard [*d*6]-cholesterol. Saponification was carried out with ethanolic KOH (2 M, 2 mL) at 80°C for 60 min. Mixture was centrifuged for 5 min at 3000 × *g* and 4°C after the addition of deionized water (2 mL) and *n*-hexane (3 mL). The upper hexane layer was collected and its 3 μL injection was analyzed using a mobile phase of acetonitrile/methanol (99:1, v/v) at flow rate of 600 μL/min. Temperature of ion source was adjusted at 300°C accompanied with declustering potential of 75 V. Data obtained was analyzed using ANALYST (1.5) software and graphs were plotted showing the quantities of phytosterol contents.

## Results

**FIGURE 1:**
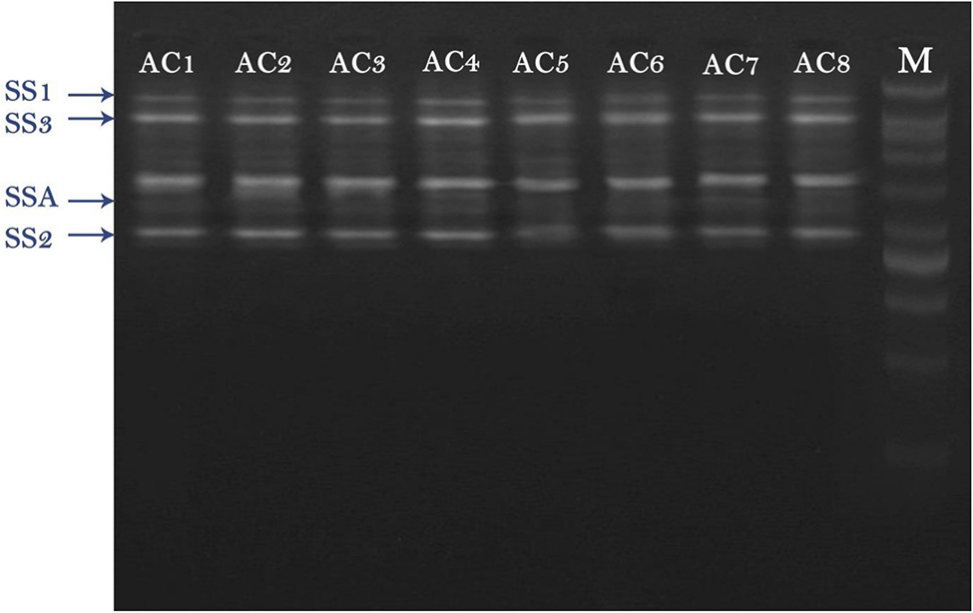
**Agarose gel image of RT-PCR product of four squalene synthase (SS) genes (i.e., SSA, SS1, SS2, and SS3) detected in Barley plants.** Barley was treated with eight bacterial strains (i.e., AC1, AC2, AC3…. AC8) to evaluate their effect on expression of SS genes. DNA Marker (DNA Ladder) was loaded in a well to compare it with other bands as a standard reference.

RT-PCR results revealed that total four types of SS genes were expressed in barley (i.e., SSA, SS1, SS2, and SS3). All these four genes exhibited different expression levels. AC8 was screened out as the best bacterial inducer, which showed maximum activity for elevation of gene expression and phytosterol contents. The most expressive gene recorded in treatment AC8 was SSA, which showed its maximum role in production of SS, and concomitantly in phytosterols production. Furthermore, SS2 was screened out as a second highly induced gene by applying treatment AC8. SS3 was screened out as the best induced gene at third level. SS1 was reported as the least expressive gene. The most favorable bacterial strain which exhibited the best relationship with barley and reported maximum gene expression was AC8. AC7 was reported as the least significant bacterial strain in expression of gene SS1 (**Figure 1**).

The maximum expression of gene SSA was recorded in treatment AC8 (174.521 ng/5 μL). AC1 and AC3 were closely related in upregulating expression of gene SSA with numeric values of 144.642 ng/5 μL and 140.248 ng/5 μL, respectively. Strain AC4 (133.511 ng/5 μL) exhibited insignificant induction difference of gene SSA in comparison to the strain AC3. However, gene SSA treated with strain AC7 (113.299 ng/5 μL) recorded significant transcriptional difference with treatment AC4; while strains AC2 and AC6 possessed insignificant difference in upregulation of gene SSA (108.95 ng/5 μL and 104.218 ng/5 μL, respectively), which was very close to the induction of the same gene by AC7. Furthermore, strain AC5 gave the least expression of gene SSA (89.278 ng/5 μL).

**FIGURE 2:**
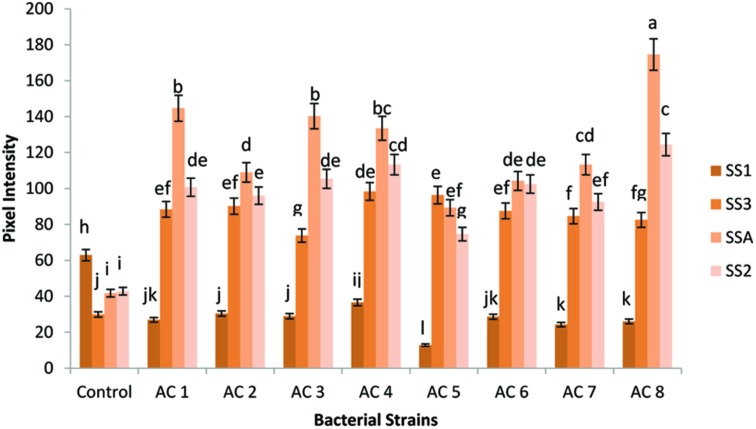
**Pixel intensity based graph of Agarose gel electrophorated with RT-PCR products of SS genes.** Expression of four SS genes (i.e., SSA, SS1, SS2, and SS3) was quantified using GELANALYZER (Lazar, Hungary) and recorded data were analyzed using DSAASTAT (Onofri, Italy). Analysis of Variance (ANOVA) and Duncan’s Multiple Range Test (DMRT) were performed at *p* ≤ 0.05, and significance of data was mentioned using alphabets (a, b, c……) over each data bar in graph, whereas error bars show standard error of each data bar.

Gene SS2 was at second position which showed more transcriptional rate than gene SS3 and SS1. However, gene SS3 stood third among the expression hierarchy of the tested genes, while gene SS1 showed the least expression than all other tested genes, i.e., SSA, SS2, and SS3 (**Figure 2**).

Squalene synthase2 gene showed the highest expression (124.4303 ng/5 μL) in bacterial treatment AC8. Gene SS2 under application of treatment AC4 (113.299 ng/5 μL) recorded insignificant difference with respective gene expression under treatment AC8. The bacterial treatments AC3, AC1, and AC6 showed insignificant differences in SS2 gene expression with transcriptional intensity of 105.39 ng/5 μL, 100.703 ng/5 μL, and 102.46 ng/5 μL, respectively. Treatments, AC2 and AC7 exhibited slight variation in upregulation of gene SS2 (96.016 ng/5 μL and 92.501 ng/5 μL). Least SS2 gene expression among eight tested bacterial strains was recorded in AC5 (89.27 ng/5 μL).

Gene SS3 exhibited maximum expression (98.359 ng/5 μL) in treatment AC4, while treatment AC5 occupied second position in this hierarchy (96.309 ng/5 μL). Bacterial strains AC2, AC1, and AC6 were found closely related in upregulation of the expression of gene SS3 (i.e., 90.157 ng/5 μL, 88.4 ng/5 μL, and 87.521 ng/5 μL, respectively). Transcription of gene SS3 was insignificantly variable under the influence of treatments AC7 (84.592 ng/5 μL) and AC8 (82.541 ng/5 μL). Treatment AC3 showed the least induction of gene SS3 (73.753 ng/5 μL) than other tested treatments, i.e., AC8, AC7, AC6, AC1, AC2, AC5, and AC4.

Squalene synthase1 gene showed the maximum expression (36.551 ng/5 μL) under treatment AC4. Strains AC2 and AC3 exhibited more or less identical patterns for induction of gene SS1, because the numeric values of gene expression (30.4 ng/5 μL and 28.935 ng/5 μL, respectively) were very close to each other. Similarly, strains AC6 and AC1 recorded almost equal rates of gene SS1 induction (i.e., 28.642 ng/5 μL and 26.885 ng/5 μL, respectively). Moreover, AC8 and AC7 also showed identical activity for inducing SS1 gene expression (i.e., 26.006 ng/5 μL and 24.248 ng/5 μL, respectively). Treatment AC5 recorded lower induction rate of gene SS1 (12.842 ng/5 μL) than induction rate of respective gene triggered by treatment AC4. Treatment AC8 was screened as the best inducer bacterial strain for SS genes of barley, which showed the highest upregulation of gene SSA and the least upregulation of gene SS1 (**Figure 2**).

Among eight bacterial strains, AC8 caused maximum elevation in transcriptional rate of gene SSA (318.51%), whereas, strain AC1 was the second best inducer for percentage change in expression of gene SSA (246.86%). Strain AC3 (236.3286%) followed the strain AC1 with insignificant difference. However, strain AC4 (220.17%) showed significant difference with strain AC7 (171.7%) and strain AC2 (161.16%) in terms of gene SSA expression. Whereas, the bacterial strains AC7 and AC2 were insignificantly different from each other. Strain AC6 exhibited 149.92% change in expression of gene SSA. Furthermore, strain AC5 caused the least change in expression percentage of the gene SSA (114.09%) than other bacterial strains. Gene SSA showed its expression against all eight bacterial treatments, but with different transcriptional rates.

Change in expression of gene SS3 was maximum (228.84%) in plants treated with strain AC4 followed by strain AC5 with insignificant difference (221.99%). Treatment AC2 upregulated the expression of gene SS3 up to 201.42%, which was insignificantly higher than the upregulation caused by strain AC6 (192.61%). Strains AC7 and AC8 recorded almost equal rates of induction of gene SS3 with numeric values of 182.82 and 175.96%, respectively. Hence, treatment AC3 exhibited the least percentage induction value of gene SS3 (146.58%).

The highest upregulation in gene SS2 was caused by strain AC8 with numeric value of 190.45%. Treatment AC4 was next in boosting transcriptional rate of gene SS2 (164.47%). Furthermore, strain AC3 elevated the transcription of gene SS2 up to 146.00%, closely followed by strain AC4. Strains AC6 and AC1 enhanced the transcription of gene SS2 with more or less equal rates of 139.16 and 135.06%, respectively. Strain AC7 elevated the transcription of the gene SS2 up to 115.92% making strain AC5 as the least efficient inducer of the gene (74.21%). Gene SS2 also showed the same variable trend of increased transcription under the influence of different treatments.

**FIGURE 3:**
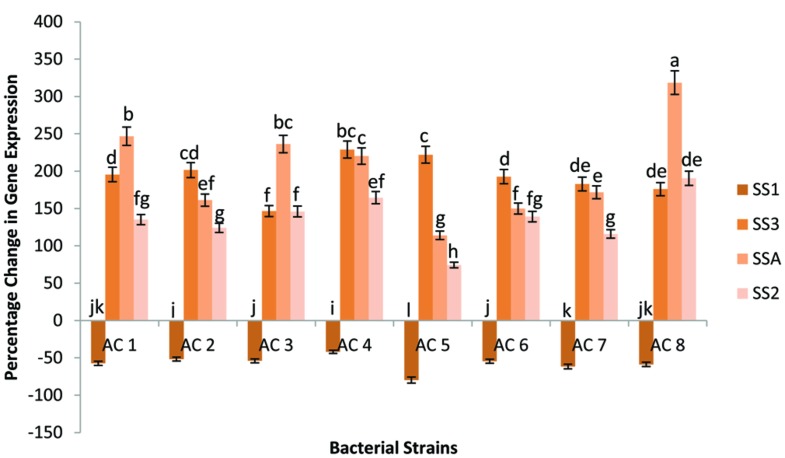
**Percentage change in expression of four SS genes (i.e., SSA, SS1, SS2, and SS3) due to application of eight bacterial strains (AC1, AC2, AC3…… AC8).** Data were statistically analysed through ANOVA and DMRT at *p* ≤ 0.05 using DSAASTAT (Onofri, Italy). Error bars show the *SE* calculated against each bar and significance of data has been mentioned through alphabets (e.g., a, b, c……) calculated at *p* ≤ 0.05.

Transcriptional rate of gene SS1 did not elevated by any of the eight bacterial strains. Gene SS1 had not shown percentage elevation of its expression in any of the eight bacterial strains. Thus, it was recoded that percentage change of expression of gene SSA was maximum under influence of strain AC8 (**Figure 3**).

**FIGURE 4:**
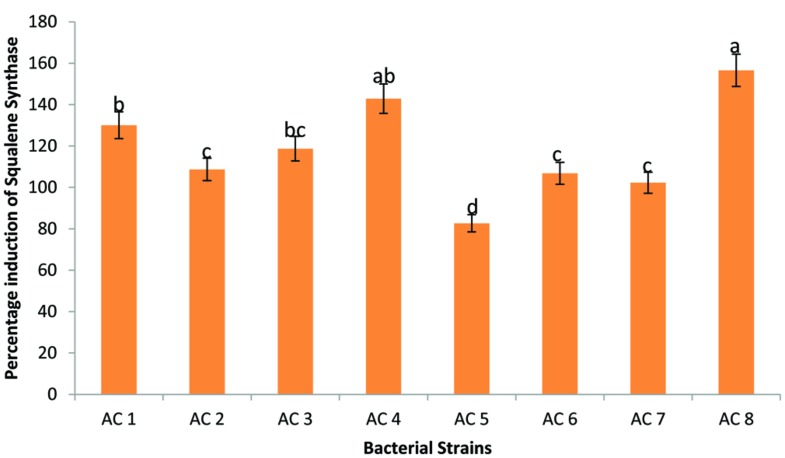
**Efficiency of eight bacterial strains (i.e., AC1, AC2, AC3…. AC8) to induce transcription of SS in Barley plants.** Data were statistically analysed through ANOVA and DMRT at *p* ≤ 0.05. Statistical analyses were performed at *p* ≤ 0.05 using Add-on package of MS-Excel, DSAASTAT (Onofri, Italy).

Maximum expression enhancement of SS genes (156.56%) was recorded due to application of treatment AC8. Strain AC4 triggered insignificantly lower expression (142.89%) of SS genes than strain AC8. Whereas, strains AC1 and AC3 were third among Squalene inducers’ index due to insignificantly different upregulation rates of 130.05 and 118.72%, respectively. Strains AC2, AC6, and AC7 showed significantly lower induction of SS genes product than strain AC3 with insignificant difference among them (i.e., 108.75, 106.80, and 102.24%, respectively). The least elevation in quantity of SS genes product was recorded due to application of treatment AC5 with numeric value of 82.67% (**Figure 4**).

**FIGURE 5:**
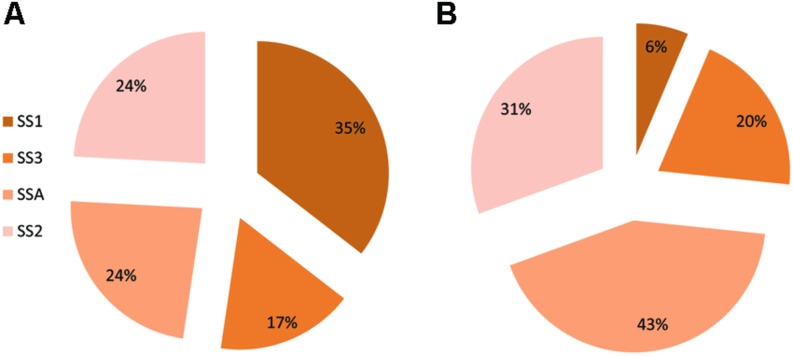
**Percentage share of SS genes (i.e., SSA, SS1, SS2, and SS3) in synthesis of total Squalene in Barley.** Squalene share was estimated in control treatment of Barley **(A)** and in Barley plants treated with bacterial strain AC8 **(B)**. Statistical package DSAASTAT (Onofri, Italy) was used to analyze data.

In control treatment of barley, plants showed varied expression of SS genes. The most expressed gene was SS1 (35%) followed by equally expressed genes of SS2 and SSA, each having a share of 24%. Gene SS3 recorded the least percentage transcriptional share of 17%. Plants treated with strain AC8 exhibited gene SSA occupying maximum percentage share of SS (43%) in its transcriptional profile. Second most expressed gene was SS2 possessing 31% share of total SS transcription. Gene SS3 occupied the third position among transcriptional index of all the tested SS genes with its share of 20%. However, gene SS1 was the least expressed gene (6%) among all SS genes. Therefore, gene SS1 possessed the maximum expression (35%) and gene SS3 showed the least (17%) in control treatment. Whereas, gene SSA recorded the highest transcriptional share of 43% and gene SS1 recorded the least share 6% among treated plants (**Figure 5**).

### Molecular Identification

**FIGURE 6:**
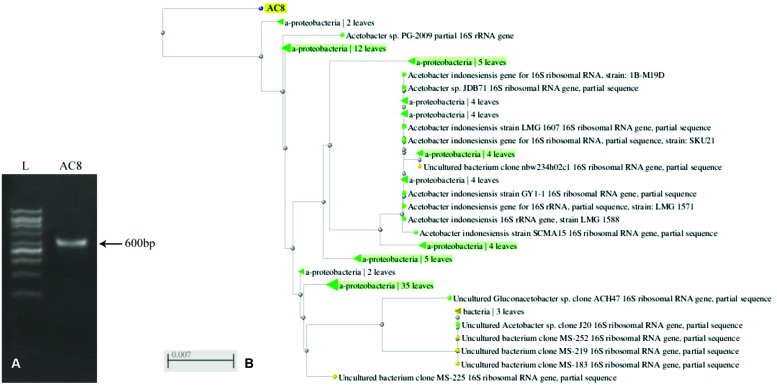
**PCR amplification of 16S sequence of *Acetobacter aceti***(A)**, and its dendrogram in comparison to other reported sequences of the same bacterial species based upon similarity index **(B)**.** Values mentioned against each strain show the phylogenetic distances of different bacterial strains.

Ribotyping of bacterial species generated a sequence of 600 bases which was submitted to GenBank under the accession number # KR024029 (**Figure 6A**). BLAST analysis of the sequence showed ≥97% similarity with previously reported sequences of *A. aceti* (**Figure 6B**). The sequence recorded the maximum similarity with strains ZIM B043, LMG-28092, and LMG-27543 of *A. aceti* (Accession # AJ012542.1, KJ147512.1, and HG424426.1, respectively).

### Verification of Phytosterol Contents

**FIGURE 7:**
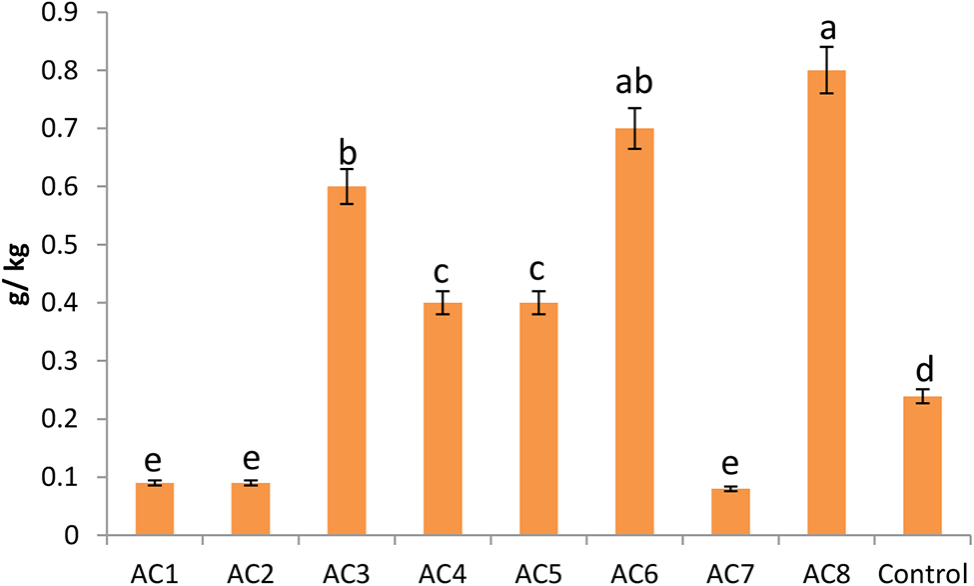
**Phytosterol contents of barley seeds harvested from barley plants treated with different bacterial treatments (i.e., AC1, AC2, AC3… AC8).** Error bars show the standard error of data bar. However, e.ach change in alphabet represents the significant difference among data bars calculated by DMRT at *p* ≤ 0.05, using statistical package DSAASTAT (Onofri, Italy).

The highest level of phytosterol contents was found in plants treated with bacterial strain AC8, which was more than three times of the contents of control plants. Strain AC6 induced phytosterols with insignificant difference from AC8. However, barley seeds harvested from AC3 treated plants recorded significantly lower phytosterol contents than AC8. Strain AC4 and AC5 showed almost equal phytosterol induction. Similarly, barley seeds harvested from strain AC1, AC2, and AC7 treated plants also exhibited almost similar phytosterol quantities, which were even lower than the control treatment (**Figure 7**).

## Discussion

Bacterial treatments affect physiological metabolism going on inside plant body (Schallau and Junker, 2010; Ahemad and Kibret, 2014; Lau et al., 2014). Commonly, they can change the rates with which genes are being transcribed inside plant cells. Transcriptional rates modify nutritional contents of commodities, thus modulating nutritional composition of plant produce (Georgieva et al., 2000; Duan et al., 2013; Ruzanski et al., 2013). Therefore, composition of plant produce can be modified by application of specific bacterial treatments. In this way, quality of plant produce can be improved by directing transcription of genes in a specific direction. Current study describes the modulation of transcriptional rates of SS genes in order to improve phytosterol contents of barley. Thus, the investigation strengthens all previous reported studies in this direction and concluded bacterial isolates which may be potentially used to improve phytosterol contents in routine diet.

Transcription of gene SS1 was decreased after application of *A. aceti*, and this trend was more or less identical in all bacterial applications. It sets a generalized notation that gene SS1 is negatively correlated with external bacterial applications. However, the reason behind these findings is not known and a number of efforts are needed to discover factors behind it. This conclusion will be helpful to understand genetics behind squalene synthesis and phytosterol production. Transcription of three squalene production genes, i.e., SS2, SS3, and SSA were upregulated after application of *A. aceti*, but the pattern and extent of enhanced transcriptional rates were greatly variable. No significant pattern could be constructed between the bacterial treatments and transcriptional elevations of SS genes. It indicated the complexity of this aspect in plant genetic studies. Therefore a number of studies should be performed to understand transcriptional responses of SS genes after bacterial treatments.

Specific genes have a definite generalized response toward bacterial applications. Response intensity of a specific gene may vary due to different types of bacterial applications but direction will never be opposite. Gene SSA was found to be more responsive toward external application of bacterial treatments revealing that it was transcriptionally more sensitive. SS2 stood second with reference to transcriptional sensitivity closely followed by SS3. It concludes that SSA has strong association with transcriptional mediators triggered by external applications (Armengaud et al., 2010; Beg et al., 2012). However, other tested genes showed weaker associations with external bacterial treatments.

Percentage share of SS1 in total squalene contents was significantly decreased in barley produce. It means that transcription of SS genes was increased with possibly enhanced production of phytosterol contents; but the effects of this altered transcription on chemical composition of phytosterols and their proportion were unknown. Identification of bacterial species was carried out as *A.aceti* (FCBP-537). *A. aceti* has never been involved in economically important plant and human diseases. Therefore, it would be appropriate to use *A. aceti* in agricultural fields to elevate the transcription of SS genes. However, application of intact bacterial species may cause unknown interactions with untargeted microbes in the surrounding environment. Therefore, active metabolites from the bacterial species must be studied and active metabolic compound/s should be identified, which is/are responsible for enhancement of transcriptional rate of SS genes in barley.

Transcriptional processes going on inside plant cells are very complex in nature. Most of the times, pattern recorded in actual biochemical quantifications strictly obeys the pattern prevailed in transcriptional data. However, in rare cases it is not true as well. Therefore, the quantification of actual phytosterol contents was performed and it was recorded that highest quantities of phytosterols were present in seeds of AC8 treated plants. It is important because AC8 also recorded the maximum induction of SS genes during transcriptional analysis. Moreover, strain AC7 showed the least induction of SS genes, and it also recorded the least quantities of phytosterols in barley seeds. Hence, the study proved a strict association between SS gene transcriptional rates and actual phytosterol contents. This finding comes in agreement with Lee et al. (2004).

*Acetobacter aceti* does not have any previous history of being a human pathogen, and it is a very important bacterial species with high use in industries (Zahoor et al., 2006; Awad et al., 2012). Current study also demonstrated *A. aceti* as a biological treatment enhancing SS transcription leading to the augmentation of phytosterol contents in food commodities. *A. aceti* does not cause any infection on humans but some researchers mistakenly described its association with pink disease of pineapple (Buddenhagen and Dull, 1967; Rohrbach and Pfeiffer, 1976). However, it has been proved through numerous studies that particular species has no involvement in pink disease of pineapple (Cha et al., 1997; Pujol and Kado, 1999, 2000; Chotani et al., 2000; Silberbach et al., 2003; Marín-Cevada et al., 2006). Thus, its application in agricultural areas including pineapple cultivation has no threat to agricultural crops and hence strongly recommended as field application.

## Conflict of Interest Statement

The authors declare that the research was conducted in the absence of any commercial or financial relationships that could be construed as a potential conflict of interest.
